# Enablers and barriers to rotavirus vaccine coverage in Assam, India- A qualitative study

**DOI:** 10.1016/j.jvacx.2024.100479

**Published:** 2024-03-19

**Authors:** Rashmi Mehra, Arindam Ray, Sabita Das, Biman Kusum Chowdhury, Seema Singh Koshal, Rhythm Hora, Amrita Kumari, Amanjot Kaur, Syed F. Quadri, Arup Deb Roy

**Affiliations:** a John Snow India, New Delhi, India; b Bill and Melinda Gates Foundation, New Delhi, India; c Government of Assam, Guwahati, India

**Keywords:** Healthcare, Vaccine, Coverage, India, Rotavirus vaccine, Universal Immunization Programme

## Abstract

**Background:**

Estimates suggest that 78,000 children died due to rotavirus gastroenteritis annually between 2011 and 2013 in India. The north eastern state of Assam reported 38.4% pediatric diarrheal admissions testing positive for rotavirus. Rotavirus vaccine (RVV) was introduced in Assam in 2017 following which the National Family Health Survey-5 (NFHS-5) (2019) revealed low RVV coverage in Assam with wide variation between the districts. the current study was conceptualized and undertaken to capture the enablers and barriers to RVV coverage in Assam.

**Methods:**

Qualitative study conducted in 5 randomly selected districts in Assam. Participants (key informants) were recruited by purposive sampling at each level of the health system including healthcare officials, service providers and caregivers based on availability. Thirty-five in-depth interviews (IDIs) and five focus group discussions (FGDs) were conducted. Interviews were tape recorded and transcribed. Data was coded and analyzed using the thematic framework approach.

**Results:**

Findings from the qualitative data collection were collated and analyzed under 7 identified themes. Difficult terrain, limited service provider availability and no catch-up training for new recruits were some of the barriers to RVV coverage. In contrast, Information, Education & Communication (IEC) in vernacular language, RVV safety profile, development partner support and adequate RVV supply were identified as some of the enablers of RVV coverage.

**Conclusion:**

Few broad recommendations to overcome identified barriers include comprehensive inter-sectoral coordination, regular monitoring and frequent refresher training sessions. There is a need for a future study utilizing existing coverage data and larger sample size to triangulate the findings of this study.

## Background

In the pre-vaccine era, a large proportion (about 35 % to 40 %) of severe diarrhea in children younger than 5 years of age was due to rotavirus. Since the epidemiology of rotavirus infection was found to be similar in both developed and developing countries, it suggested that improved sanitation alone remained insufficient to prevent infection [1]. Rotavirus deaths accounted for almost 3.4 % of all child deaths in 2013 with a cause-specific mortality rate of 33 deaths per 100 000 children aged < 5 years. Almost two-thirds of all rotavirus associated deaths were concentrated in 10 countries with 4 countries (Democratic Republic of the Congo, India, Nigeria, and Pakistan) accounting for half of the deaths [2]. As per estimates from India from 2011 to 2013, rotavirus gastroenteritis is associated with 78,000 deaths among children annually, with about 59,000 in the under 2 years age group [3]. The North eastern state of Assam has been one of the worst affected States with 38.4 % pediatric diarrheal admissions testing positive for rotavirus in 2015–16 [4]. According to National Family Health Survey-4 (NFHS 4), 50.8 % of children with diarrhea in Assam were taken to a health facility for treatment [5].

Based on the recommendation of the National Technical Advisory Group on Immunization (NTAGI) and after approval of Mission Steering Group (MSG), states were identified for phase-wise introduction by the expert committee. The states were selected based on criteria such as diarrheal disease burden, adverse event following immunization (AEFI) preparedness, routine immunization (RI) coverage, system preparedness and states’ willingness to introduce the vaccine.

Considering the dire need, Rotavirus vaccine was introduced in Assam on 14th June 2017 under the Universal Immunization Programme (UIP). This roll out was done in Assam as part of the second phase of expansion of the RVV introduction in India. The vaccine was introduced in all 33 districts of the state comprising of 177 blocks.

Assam has made significant improvement in the coverage of vaccines administered under the UIP such as the Pentavalent and Oral Polio Vaccine. Though overall coverage has improved, inter district and intra district variations exist with some areas performing better than the others. For example, the first evaluated coverage data in NFHS-5 reveals that the RVV 3 coverage in Assam is 45.4 %, with Dhemaji recording the highest coverage of 76.7 % and Sivasagar with the lowest coverage of 30.4 % [6]. This indicates that there are multifaceted factors which impact the coverage of the rotavirus vaccine in various districts of Assam.

Owing to the low RVV coverage with wide inter-district variation highlighted by NFHS-5, the current study was conceptualized and undertaken to capture and understand the enablers and barriers to RVV coverage in Assam.

## Methods

The present study used qualitative methodology to capture the perceived factors affecting RVV coverage in Assam. Qualitative studies have been classified by some on a hierarchy of evidence ranging from single case studies through to generalisable studies [7]. The present study attempts to explore the factors (both enablers and barriers) for RVV coverage in Assam through key stakeholders (Government officials, service providers and caregivers) interviews.

**Fig. 1:**
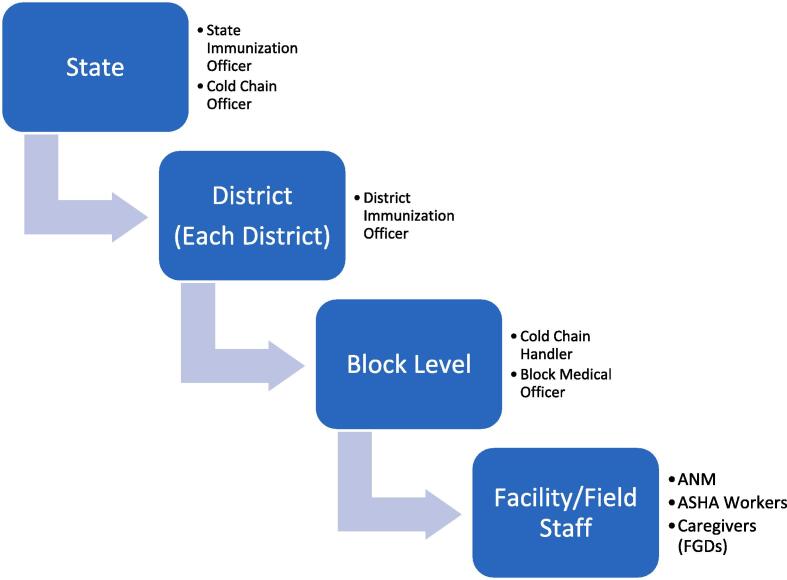
Participant recruitment under the present study.

Participants (key informants) were recruited by purposive sampling at each level of the health system including State and District officials, healthcare providers (medical officers (MOs), Auxiliary Nursing Midwife (ANMs), Accredited Social Health Activist (ASHA) workers and pool of caregivers based on availability (Fig. 1). All caregivers, ANMs and ASHAs at the respective facilities were approached. Only consenting adults (above the age of 18 years) who agreed to be a part of the study were included in the study.

**Fig. 2:**
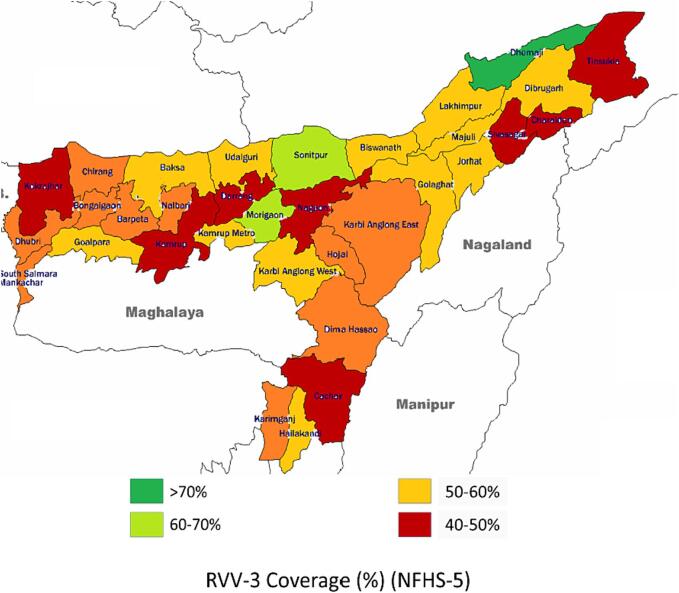
District wise RVV-3 coverage (%) in Assam as per NFHS-5.

In order to best grasp the essence of RVV introduction in Assam, a district was included from each of the administrative divisions of Assam. Following a random sampling, Hailakandi, Darrang, Kamrup Metro, Nagaon and Jorhat were included in the present case study. Although randomly selected, these 5 sample districts are potentially representative of the geographic, cultural and socio-economic diversity of Assam. Fig. 2 illustrates the RVV-2 coverage reported in different districts of Assam as per the NFHS-5. We can conclude from the same the selected districts are from varying RVV-3 coverage brackets- Hailakandi (50–60 %), Darrang (40–50 %), Kamrup Metro (50–60 %), Nagaon (40–50 %) and Jorhat (50–60 %) [6].

Specially constructed interview guides were used each at 5 different levels- state (State Immunization Officer (SIO) and Cold Chain Officer (CCO)), district (District Immunization Officer (DIO)), planning unit (MO), and sub-center (ANM) and community mobilizer at village level (ASHA). Interviewees were selected from the block with the highest number of RVV1 deliveries according to Health Management Information System (HMIS) data (2019–20). Structured, in-depth face to face interviews were done at a time convenient for participants between Feb 7 and Feb 14, 2021. With participant permission, all interviews were audio-recorded. Participants' age, designation, years of work experience and time since they have been posted at current location were obtained at the start of the interview. The interview guides for each group of stakeholders consisted of open-ended questions covering the following broad themes [8]:

1. Planning and processes
2. Training of Health Workers
3. Communication for demand generation
4. Vaccine uptake after the introduction
5. Supervision and monitoring
6. Data Management
7. Vaccine supply chain

Each of these areas were studied at the field level through IDIs with the DIOs, MOs, ANMs and ASHAs and FGDs with the mothers or caregivers. This exercise highlighted the key enablers that were crucial for the programme success and brought out some of the barriers that were faced in Assam.

The study objectives and voluntary nature of the study were explained to participants, and informed consent was obtained before each interview. With the consent of the participants, the interviews were audio-recorded and later cautiously transcribed verbatim to generate accurate transcripts for effective interpretation and analysis. During the transcription process, the anonymity of the participants was ensured. All the data can be made available on request. Throughout this study, we followed the Standards for Reporting Qualitative Research guidelines.

Thematic analysis of the interview transcripts was conducted using NVIVO 12 qualitative data analysis software. The analysis involved thorough and repeated reading of the transcripts followed by coding of the text. The texts were coded and re-coded under different codes. An iterative process was followed for the thematic analysis to develop a narrative capturing the enablers and barriers for RVV coverage in Assam.

Necessary approvals and permissions to undertake the study were obtained from the Assam Immunization Division, Department of Health. Informed consent was obtained from all participants before commencing the interview

## Results

The present study aimed to capture the perceived factors affecting RVV coverage in Assam through IDIs of relevant stakeholders including health officials, service providers and caregivers of beneficiaries. A total of 35 IDIs (Seven in each district) with supply side players and 5 FGDs with caregivers were conducted across the 5 included districts. The findings of the study have been collated under the identified 7 themes.

### Planning and processes

It was found that overall the process of administering RVV under the UIP was well-defined. Prior planning initiatives included timely preparation of due list of beneficiaries by the ANMs were reported by most of the participants. As highlighted by MOs at facilities, this ensured adequate amount of vaccine supply at the sessions site. Further, ANMs emphasized that line listing of all eligible children was carried out to identify beneficiaries. However, it was reported that there are many hard-to-reach areas such as “char areas” (riverine areas) and flood prone areas which are difficult to access during the rainy season and also do not have adequate health facility/infrastructure. This contributed to high drop outs as well as left outs.

Since the ANMs are primarily responsible for vaccination sessions, in an attempt to overcome repercussions of absenteeism, neighbouring ANM would facilitate the immunization session. Alternatively, the sessions were also organized on Saturdays to ensure availability of beneficiaries. Additionally, children who missed out on one or more dose of RVV were mobilized in subsequent sessions to ensure that they complete the 3-dose schedule. Still, it was reported by respondents that the workforce was not rationally distributed in hard-to-reach areas leading to excess burden on some health workers. This led to sub-optimal delivery of immunization services which hindered the efforts to improve the RVV coverage.

On enquiring about community mobilization, it was consistently found to be conducted by ASHAs by visiting all enlisted beneficiaries, two days before the scheduled Village Health Nutrition Day sessions (VHND). Few ASHAs also mentioned that reminder calls were also made to all the beneficiaries by them.

Other enablers brought forth were regular Village Health, Sanitation and Nutrition Committee (VHSNC) Meetings, consistent delivery of four key messages to every caregiver at the session site. Few respondents mentioned that they were unsure whether all 3 doses of RVV would be considered for the incentive received for full immunization coverage (FIC) by ASHAs.

*“Every sub-centre has 2 ANMs. That’s how we have grouped to ensure coverage by ANMs. NHM (National Health Mission) staff also get deputed so that it is managed well. Session dates are moved around to ensure attendance of staff.”*

-DIO

### Training of health workers

Two key aspects of the training sessions found to be helpful by all the stakeholders were exhaustive training sessions and involvement of development partners in organizing training sessions. These sessions were largely described as “very useful” by the stakeholders. Most respondents mentioned that organizations like World Health Organization (WHO), United Nations Children's Fund (UNICEF), United Nations Development Programme (UNDP), and & John Snow India (JSI) facilitated the process of training and roll out and their techno-managerial assistance and expertise were very most useful in organizing and monitoring large scale comprehensive quality trainings.

Contrastingly, some of the key stakeholders like the DIOs and MOs reported the need for refresher sessions since they were not designated in their respective positions during the launch of RVV. Some respondents (service providers) were found to be unsure about the vaccine schedule to be followed when there is a delay in age appropriate RVV doses. Further, it was found that training for few newly recruited ANMs was delayed due to the pandemic.

*“There is a need for refresher ANM training. It is needed so they know the correct schedule. Like, we need to tell them that RVV1 can be given with Penta2/OPV2 (Oral Poliovirus Vaccine)”*

-DIO

### Communication for demand generation

The respondents mentioned a number of efforts undertaken to enable communication for demand generation of RVV including house visits by ASHAs to educate the community about the benefits of the new vaccine and targeted messages to the community such as “no fear related to vaccine”, “easy availability in government facilities”, “protection against diarrhea”, and “sweet taste of the vaccine”. These reportedly helped in creating awareness, mobilization, and demand generation for the RVV. Furthermore, monthly VHND sessions and sensitization of mothers and pregnant women and display of targeted IEC materials in multiple languages at the Anganwadi centers/session sites were reported enablers. Respondents also stated that pamphlets and leaflets drafted in these languages were distributed during house visits and community meetings before the introduction of RVV which were very useful in generating demand.

On the other hand, respondents reported issues in timely mobilization by ASHAs in certain locations. Respondents also expressed the need for targeted interventions in slum areas in regional languages such as awareness through street play, puppet shows which could be delivered in the language of that particular residing population (for example: Bihari, Bengali, Assamese, and Bodo etc.). This was especially mentioned in the urban district of Kamrup Metro where residents from neighbouring states reside requiring a multi-linguistic approach for demand generation.

*“Nothing specific with RVV, however for strengthening of overall RI, ASHA needs some extra support. ASHA do not get any support from the AWW, she has to do all the line listing, due list preparation, house to house visit, bringing the beneficiaries to the session sites, conducting VHND sessions all by herself; this has led to lot of burden and extra work on her.”*

-ASHA

### Vaccine uptake after the introduction

Pre-introduction communication about vaccine roll out was highlighted as a major enabler for vaccine uptake since communication and awareness strategies influenced target behavior significantly and improved vaccine acceptance. Safety of the vaccine and positive impact of immunization was reported as another enabler as the incidence of diarrhea reduced since introduction of the vaccine, it enhanced the community’s trust in the vaccine. Vaccine hesitancy in some pockets was reported as an important barrier to RVV coverage. Few respondents mentioned reluctance to accept government vaccines and low level of trust in some minority pockets and slum-like areas which do not have a specific demarcation.

Another important barrier to RVV coverage was reported to be the sub optimal use of various media including lack of advance reminders through SMS/call for immunization dates.

A few respondents informed about the prevailing fear of opening new vials among service providers and ensuring its full usage. They mentioned that in a few instances the ANMs in these locations, did not administer the remaining beneficiaries who reached the session site 4 h after opening a vial.

Other barriers reported to uptake of RVV were migration of the family, unavailability of parents to bring child to session site and child not feeling well on the day of vaccination. These barriers were reported in the urban district of Kamrup Metro which was one of the poor performing districts of Assam as per the NFHS-5 data.

### Supervision and monitoring

It was reported by the respondents that both regular supervisory visits conducted by officials (from state and district level) and regular monitoring were important enablers for RVV coverage in Assam. Interviewees mentioned that there were approximately 8–10 supervisory visits by district officials in a month. They also mentioned that monthly review meetings organized at block level along with ANMs, ASHAs and Data managers helped identify any pockets with low immunization coverage, including RVV coverage. Few barriers to supervision and monitoring included access issues for hard-to-reach areas especially during summer/rainy season and high workload of DIOs/MOs due to involvement in other programmes.

*“There should be a dedicated immunization doctor responsible only for vaccination and immunization. Too many other duties for the MO to deal with.”*

-MO

### Data management

Respondents emphasized on data management and appropriate use of quality data in programme management and decision making. Some of the key initiatives undertaken to facilitate appropriate data management include monthly review meetings and regular analysis of data for programmatic improvement. However, most respondents mentioned issues related to inadvertent errors in data processing from a paper-based to a digital format were faced.

*“Every Wednesday after session ANM sends hard copies to LHV. Performance report are being sent during last part of the month. All CCPs (Cold Chain Points) sends reports to LHV. From respective CCP, data sent to BDM which is compiled monthly in HMIS and send it to District.”*

-MO

### Vaccine supply chain

The key enablers of vaccine supply chain were reportedly adequate supply of RVV across most of the districts and improved efficiency in supply chain through eVIN. Good coordination among stakeholders including MO, Cold Chain Handler (CCH), and Lady Health Visitor (LHV) ensures adequate vaccine supply without any stock outs was reported. Furthermore, it was reported that regular trainings were provided to the CCH on using eVIN which has reduced instances of vaccine stock-outs and helped improve availability of RVV. Additionally, respondents mentioned poor last mile delivery associated with limited financial allocation, lack of vehicles for vaccine transportation, and shortage of human resources.

*“It (PCV) is usually always available. It was not available once in 2019 for 1*–*2 weeks”*

-ANM

*“Stocks have always been smooth. We always ensure a buffer stock. Vaccine store manager is very efficient.”*

-MO

## Discussion

The present study captured enablers and barriers to RVV coverage in Assam through in-depth interviews and FGDs with key stakeholders. Based on the findings of the current study, it was found that despite continued effort at all levels of the health system, few areas require attention to improve the existing RVV coverage in Assam. An important area of concern highlighted was the need to strengthen the UIP in urban areas. Poor immunization coverage in urban areas has been highlighted in previous studies [9], [10], [11]. This disparity in coverage trend could be attributed to a focus on limiting dropouts in rural areas [10]. A potential solution could be utilization of existing MOs posted under the National Urban Health Mission (NUHM) programme for effective monitoring, supportive supervision as well as monthly review of critical elements of respective areas/wards. Additionally, the use of mobile sessions and adaptable schedules can significantly contribute to enhancing immunization efforts in these regions. Health workers should consistently compile and use lists of due children for tracking purposes. Establishing effective communication interventions through advocacy, interpersonal interaction, and community engagement can help create a ongenial environment, enabling women in urban slums to make informed decisions about immunization, ultimately reducing dropout rates [12].

Another important take away from the present study is the need for inter sectoral coordination in the immunization programme which is in agreement with previous studies [13], [14]. Literature suggests actionable points including active involvement of block/ sectoral MOs in the Immunization program i.e., in the process of micro- planning, monitoring, evaluation of poor performing health sub-centres [15], [16]. Religious leaders, influencers and media at the local level should be involved for convincing certain specific vaccine hesitant groups [16], [17], [18]. This will further enhance acceptance towards vaccination which in turn will increase demand. The study also suggests need for reiteration of information regarding ASHA incentives provided for fully immunized children.

The findings concerning data quality and management reiterate previous findings which stress on the importance of data quality and management [19], [20]. Potential solutions could include monthly reviews by concerned MO in consultation with the Block Data Manager (BDM) could be implemented followed by feedback and evaluation by District level officials. The priority should be on people, emphasizing the importance of local education and enhancing the skills of healthcare workers to minimize occurrences like data entry mistakes. It is crucial to revamp the supervision methods for healthcare workers, making them more supportive and providing them with timely and pertinent performance feedback. This kind of framework would not only establish accountability but also inspire healthcare workers to enhance the accuracy of data [21].

Additionally, there is a need to emphasize on regular monitoring and supportive supervision at all levels. Supervisory visits should be made in poor performing blocks/Sub Centres. This is in accordance to previous studies which have found supportive supervision to be complementary to other interventions for immunization coverage improvement [22], [23]. There is also a need for trainings and refresher sessions to be conducted for the newly recruited ANMs regarding UIP, including the RVV implementation to ensure efficient and effective programme implementation. This is in line with existing literature emphasizing on capacity building of front line health functionaries [24]. Constant follow up is necessary to traverse the identified gaps.

To the best of our knowledge, this is the first study to explore the enablers and barriers for RVV coverage in Assam. This study had a robust sample size of 35 which allowed us to collect rich data which could be used for the entire state of Assam. In spite of our best efforts, few limitations of the present study could not be averted. In the present study, the districts were randomly selected resulting in inclusion of districts with RVV-3 coverage levels ranging between 50 and 70 %, excluding the best performing and poor performing districts. In the future studies, it might be useful to purposively include the best and worst performing districts to gain insights into what worked for the best performing and what did not in the poor performing districts. This would allow to develop tailored strategies for each district for tackling with their challenges using best practices of another district within the same state. Since the present study was a qualitative study, a considerable dependency remains on the availability of the interviewee. Future studies can consider telephonic interviews instead of in-person interviews.**.**Undeniably, there is a need for a large-scale study in the future, for better understating of enablers and barriers for RVV coverage spanning the length and breadth of Assam while simultaneously triangulating findings with other indicators of maternal and child health. Superimposing RVV coverage with other maternal and child health indicators will help identify geographical pockets requiring focused attention.

## Conclusion

The present study aimed to capture the enablers and barriers to RVV coverage in the state of Assam through qualitative in-depth interviews of key stakeholders including officials, service providers and caregivers of beneficiaries. A number of enablers and barriers were highlighted by the respondents in each of the 7 thematic areas contributing to the coverage of vaccines. The study also provided broad overarching recommendations to overcome the identified barriers through the study. This study also highlights the need for a future study utilizing existing coverage data and larger sample size to triangulate the findings of this study.

## Ethics approval and consent to participate

The present study was conducted as a directive and in association with the Assam Immunization Division, Department of Health, Government of Assam and all methods were performed in accordance with the relevant guidelines and regulations. Necessary approvals and permissions to undertake the study were obtained from the Assam Immunization Division, Department of Health. Since the present study comes under the category of negligible risk it was found exempt of ethics approval by the Government of Assam. This was in accordance to practice followed in other countries [25]. Informed consent was obtained from all participants before commencing the interview. Only those who provided with consent were included in the study.

## Consent for publication

Not applicable. All collected data has been anonymized.

## Availability of data and materials

The datasets used and/or analyzed during the current study are available from the corresponding author on reasonable request.

## CRediT authorship contribution statement

**Rashmi Mehra:** Investigation, Methodology, Writing – original draft, Writing – review & editing. **Arindam Ray:** Conceptualization, Investigation, Resources, Writing – review & editing. **Sabita Das:** Investigation, Methodology, Project administration, Resources. **Biman Kusum Chowdhury:** Investigation, Methodology, Project administration, Resources. **Seema Singh Koshal:** Investigation, Methodology, Resources, Writing – original draft, Writing – review & editing. **Rhythm Hora:** Data curation, Formal analysis, Visualization. **Amrita Kumari:** Supervision, Visualization, Writing – original draft. **Amanjot Kaur:** Data curation, Formal analysis, Writing – original draft. **Syed F. Quadri:** Investigation, Methodology, Project administration, Writing – review & editing. **Arup Deb Roy:** Conceptualization, Validation, Visualization, Writing – original draft, Writing – review & editing.

## Funding

This work was supported by the Bill and Melinda Gates Foundation (INV-030655).

## Declaration of competing interest

The authors declare that they have no known competing financial interests or personal relationships that could have appeared to influence the work reported in this paper.

## Data Availability

Data will be made available on request.
